# Hyperglycemia at admission is a strong predictor of mortality and severe/critical complications in COVID-19 patients: a meta-analysis

**DOI:** 10.1042/BSR20203584

**Published:** 2021-02-10

**Authors:** Yan Yang, Zixin Cai, Jingjing Zhang

**Affiliations:** National Clinical Research Center for Metabolic Diseases, Metabolic Syndrome Research Center, Key Laboratory of Diabetes Immunology, Ministry of Education, and Department of Metabolism and Endocrinology, The Second Xiangya Hospital of Central South University, Changsha 410011, Hunan, China

**Keywords:** Complications, Coronavirus Disease 2019, Glucose, Hyperglycaemia, Mortality, SARS-CoV-2

## Abstract

**Background:** Hyperglycemia at admission has been demonstrated to exacerbate the outcomes of coronavirus disease 2019 (COVID-19) but a meta-analysis is lacking to further confirm this hypothesis. The purpose of this meta-analysis was to summarize the evidence on the association between hyperglycemia at admission and the development of COVID-19. **Method:** Four databases namely, PubMed, Web of Science, Embase and Cochrane Library, were screened for eligible studies. STATA software was utilized to pool data for this meta-analysis. The primary outcomes included mortality and severity. Odds ratios (ORs) and 95% confidence intervals (CIs) were calculated with random-effects models, and the quality of evidence was appraised by the Newcastle–Ottawa Scale (NOS). This meta-analysis was prospectively registered online on PROSPERO, CRD42020191763. **Results:** Sixteen observational studies with 6386 COVID-19 patients relating hyperglycemia at admission to COVID-19 outcomes were included. The overall data demonstrated that, compared with the control, the hyperglycemia at admission group was more likely to have increased mortality (OR = 3.45, 95% CI, 2.26–5.26) and severe/critical complications (OR = 2.08, 95% CI, 1.45–2.99) of COVID-19. **Conclusion:** Hyperglycemia at admission in COVID-19 patients may be a strong predictor of mortality and complications.

## Introduction

Since the first diagnosed case in December 2019, coronavirus disease 2019 (COVID-19), which is caused by severe acute respiratory syndrome coronavirus 2 (SARS-CoV-2), has become a raging global pandemic [1,2]. As of 17 January 2021, 95007057 cases have been reported, with 2032106 deaths in total according to worldwide statistics [3]. Remdesivir, the drug for COVID-19 approved by the FDA [4], can reduce the length of stay (LOS) to some extent [4]; a few anti-SARS-CoV-2 vaccines, such as the mRNA COVID-19 vaccine (mRNA-1273) from Moderna and the non-replicating adenovirus type 5 (Ad5)-vectored COVID-19 vaccine, have been announced with high success rates [5,6], but the pandemic is still raging [7]. Given that the epidemic of COVID-19 is fast and unresolved, it is urgently necessary to identify the important features for COVID-19 to achieve better control of the COVID-19 pandemic.

Recently, hyperglycemia has been demonstrated to be related to adverse outcomes in COVID-19 patients [8,9]. Several observational studies have provided clinical evidence that uncontrolled hyperglycemia may lead to a longer LOS and significantly higher mortality in COVID-19 patients [10,11]. Additionally, poor glycemic control in COVID-19 patients without diagnosed diabetes may still contribute to a higher risk of poor outcomes and death [10]. Considering that the causality between hyperglycemia and the outcome of COVID-19 has not been determined in a previous meta-analysis [12], we further conducted the present study to gather evidence on the association between hyperglycemia at admission and the development of COVID-19 to prove the predictive role of hyperglycemia in COVID-19.

In the present study, we conducted a systematic review and meta-analysis to investigate the association between hyperglycemia at admission and outcomes of COVID-19. Our findings indicated that hyperglycemia at admission predicts increased decease severe/critical complications and higher mortality, implicating that hyperglycemia at admission may pose a possible prognostic factor in COVID-19 patients. These findings indicated that poor glucose control may be a negative factor for poor outcomes or disease progression in COVID-19 patients. As there are many people who suffer from both COVID-19 and diabetes or underlying hyperglycemia, more attention should be paid to glucose control in patients hospitalized for COVID-19.

## Methods

This meta-analysis was performed strictly in accordance with the PRISMA (Preferred Reporting Items for Systematic Reviews and Meta-Analyses) statement guidelines as described previously [13].

### Article search strategy

We searched the four databases, namely, PubMed, Web of Science, Embase and Cochrane Library, to find eligible studies. Searches for all published articles relevant to glucose control and COVID-19 were performed. The following search items were used: ‘glucose control’, ‘glucose’, ‘glycemic’, ‘HbA1c’, ‘coronavirus’, ‘SARS’, ‘2019 novel coronavirus’, ‘COVID-19’ and ‘SARS-CoV-2’. Additional papers were identified by manually searching and citation tracking.

### Selection criteria

Two reviewers (Y.Y. and Z.C.) independently reviewed all the included studies, and the determination of eligible studies was finalized. Disagreements were solved through negotiation or the help of a third reviewer (J.Z.). The inclusion criteria of the present paper are as follows: (1) studies with information on hyperglycemia and outcomes of COVID-19, including mortality, severity or complications of COVID-19 and (2) studies that measured or classified hyperglycemia at admission. Articles were excluded if they met the following criteria: (1) studies that did not have available information or usable data for the aim of this meta-analysis and (2) reports published as letters, reviews, editorials or conference abstracts.

### Data extraction

Two authors (Y.Y. and Z.C.) imported relevant articles into EndNote X9 software and reviewed them independently. Discrepancies between authors were settled with the help of a third reviewer (J.Z.). The following information was extracted by two independent investigators from the selected studies and then imported into Excel software: author, year, country, type of study, age, sample size, population, indicators of blood glucose control and COVID-19 outcomes.

### Quality assessment of studies

The quality of the eligible studies was assessed by the Newcastle–Ottawa Scale (NOS) [14]. We assessed the quality of all relevant studies on the basis of the type of study, sample size, participant selection, representativeness of the sample, adequacy of follow-up, comparability (exposed–unexposed or case–control), and method of ascertaining cases and controls. A study with a score of 6 or more was categorized as a high-quality study.

### Statistical analysis

All analyses were performed using Stata (Version 13.0). The results regarding the correlation between glycemic control and adverse outcomes were expressed as pooled odds ratios (ORs) and 95% confidence intervals (CIs). OR values > 1 represent a direct association, and those OR values < 1 represent an inverse association. All results of the included studies were calculated with random-effects models. *I^2^* statistics were used to assess the degree of heterogeneity: less than 25% represented low heterogeneity, while 50 and 75% were the cut-offs of moderate and high degrees of heterogeneity, respectively. Begg’s and Egger’s tests and funnel plots were used to detect potential publication bias, with a *P*-value <0.05 suggesting the presence of bias. The trim-and-fill method was also performed to obtain an adjusted effect size when encountering publication bias. The leave-one-out method for sensitivity analysis was performed to assess whether the results were sufficiently robust.

## Results

### Search results

**Figure 1:**
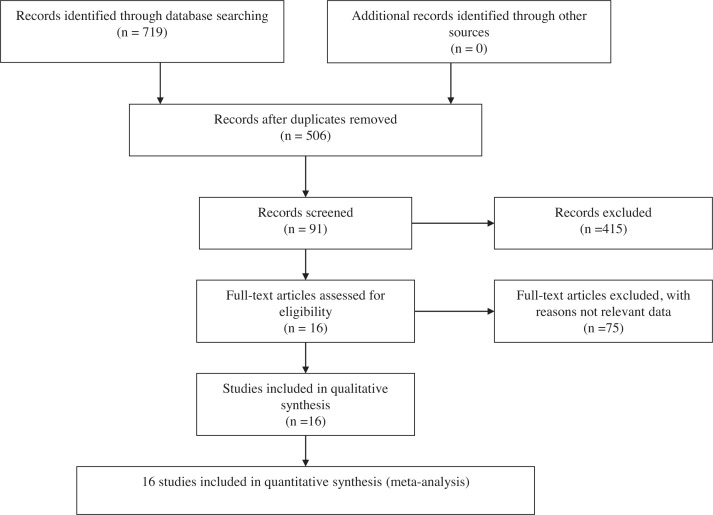
Flow diagram of the study selection process

A total of 719 initial studies were available after search, from which 213 duplicate studies were excluded. According to the title and abstract screening, 415 articles were eliminated and 91 articles remained. A total of 75 articles were excluded on the basis of the full text. Finally, 16 studies [8–10,15–26] with 6386 COVID-19 patients were eligible and included in the meta-analysis. The process of study selection with reasons for exclusion is shown in Figure 1.

### Study characteristics

The basic characteristics of the studies and results of the quality assessment by NOS are shown in Table 1. Among the 16 studies included in this analysis, most (12/16) were performed in China, 3 were performed in Italy, and 1 was performed in the U.S.A. (Table 1). The publication year of all 16 included studies was 2020. Additionally, the definition of hyperglycaemia and the methods for glucose measurement in each included study are exhibited in Table 2.

**Table 1:** Description of eligible studies reporting the association between hyperglycemia at admission and the outcomes of COVID-19

No	Author	Year	Country	Age (years)	Type of study	Sample size	Population	NOS
1	Bode	2020	U.S.A.	Control: 61; hyperglycemia: 65	A retrospective observational study	1122	COVID-19 patients	7
2	Sardu	2020	Italy	Control: 66.6 ± 11.5; hyperglycemia: 68.5 ± 5.8	ND	59	COVID-19 patients with moderate disease	8
3	Marfella	2020	Italy	Control: 66.6 ± 12.2 hyperglycemia: 65.7 ± 13.4	Short report	475	Tocilizumab therapy in COVID-19 patients	6
4	Jianfeng Wu	2020	China	62.0 (50.0, 70.0)	A retrospective cohort study	2041	COVID-19 patients	9
5	Xi Jin	2020	China	46.14 ± 14.19	ND	74	COVID-19 hospital patients with gastrointestinal symptoms	8
6	Huiqing Li	2020	China	61 (49, 68)	A retrospective study	453	COVID-19 patients	8
7	Xiaohui Liu	2020	China	60.45 ± 11.51	ND	88	COVID-19 patients	7
8	Yan Zhang	2020	China	64 (56, 70)	A retrospective cohort study	258	COVID-19 patients	9
9	Yang zhang	2020	China	62.7 ± 14.2	A single-center, retrospective, observational study	166	COVID-19 patients	8
10	Sufei Wang	2020	China	59.0 (47.0, 68.0)	A multicentre retrospective study	605	COVID-19 patients	8
11	Yumin Li	2020	China	65 (57, 71)	A retrospective cohort	132	COVID-19 patients	9
12	Coppelli	2020	Italy	ND	A retrospective study	271	COVID-19 patients	7
13	Guozhen Li	2020	China	63 (50, 75)	The retrospective study	199	COVID-19 patients	8
14	Ming Deng	2020	China	18–40	A retrospective study	65	Young COVID-19 patients	9
15	Sheng-ping Liu	2020	China	64 (24, 92)	The retrospective study	255	COVID-19 patients	8
16	Qin Liu	2020	China	68.0 (61.0, 78.0)	A multicenter, retrospective, cohort study	123	COVID-19 patients	9

Abbreviation: ND, not determined.

**Table 2:** Description of definition of hyperglycemia and method of glucose measure

No	Author	Definition of hyperglycemia	Glucose measure
1	**Bode**	Uncontrolled hyperglycemia was diagnosed when two or more BGs > 180 mg/dl occurred within any 24-h period at admission	Random blood glucose level
2	**Sardu**	Admission glycemia > 7.77 mmol/l	Fasting blood glucose level
3	**Marfella**	Admission blood glucose levels ≥ 140 mg/dl	Fasting blood glucose level
4	**Jianfeng Wu**	Admission glycemia ≥ 6.1 mmol/l	Fasting blood glucose level
5	**Xi Jin**	ND	ND
6	**Huiqing Li**	fasting glucose 5.6–6.9 mmol/l and/or HbA1c 5.7–6.4%	Fasting blood glucose level
7	**Xiaohui Liu**	ND	ND
8	**Yan Zhang**	ND	Fasting blood glucose level
9	**Yang Zhang**	No diabetes history, FPG levels ≥7.0 mmol/l once and HbA1c values < 6.5%	Fasting blood glucose level
10	**Sufei Wang**	FBG ≥ 7.0 mmol/l at admission	Fasting blood glucose level
11	**Yumin Li**	Admission glucose > 11 mmol/l	Random blood glucose level
12	**Coppelli**	Admission glucose ≥ 7.78 mmol/l	Fasting blood glucose level
13	**Guozhen Li**	FBG at admission	Fasting blood glucose level
14	**Ming Deng**	Elevated FBG level	Fasting blood glucose level
15	**Sheng-ping Liu**	Admission FBG ≥ 7 mmol/l	Fasting blood glucose level
16	**Qin Liu**	Admission FBG ≥ 6.5 mmol/l	Fasting blood glucose level

Abbreviation: ND, not determined.

### Hyperglycemia at admission and mortality of COVID-19 patients

**Figure 2:**
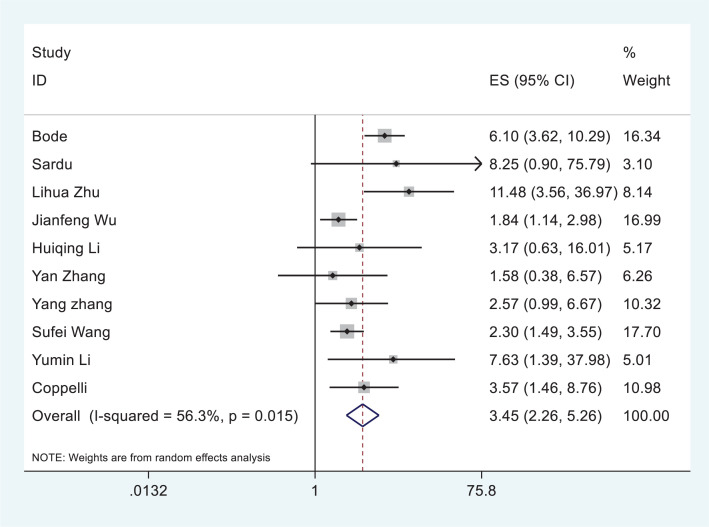
Forest plots of ORs for the association between hyperglycemia at admission and mortality of COVID-19

Ten studies have reported the effects of glucose control on COVID-19 mortality. Compared with that in the control group, the overall risk of mortality in the hyperglycemia at admission group was significantly higher (OR = 3.45, 95% CI, 2.26–5.26, *I^2^* = 56.3%, *P*=0.015) (Figure 2). Neither Egger’s regression test (*P*=0.303) nor Begg’s test (*P*=0.375) demonstrated any possibility of publication bias. Funnel plots also revealed a symmetric distribution with no publication bias among the studies (Supplementary Figure S1). Sensitivity analysis performed by removing one study each time did not alter the results materially (Supplementary Figure S4), indicating that these results were stable.

### Hyperglycemia at admission and severe/critical complications of COVID-19

**Figure 3:**
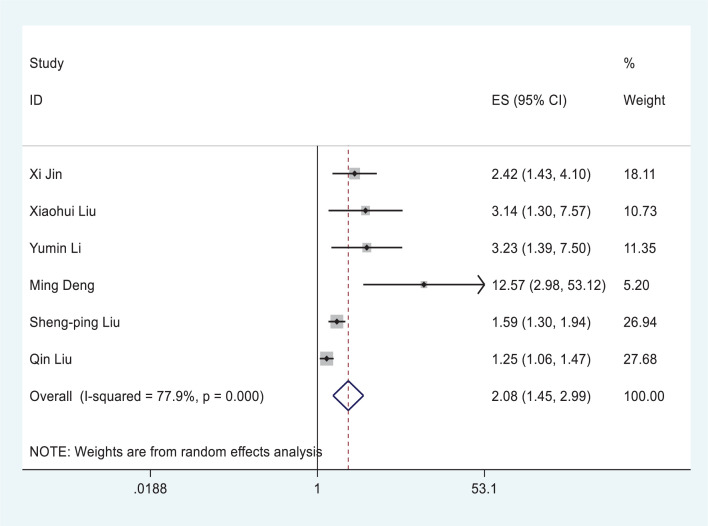
Forest plots of ORs for the association between hyperglycemia at admission and severe/critical complications of COVID-19

The overall analysis included six studies which reported quantitative data on severe/critical COVID-19 illness. All extracted data were divided into severe/critical illness according to the standard of illness severity level defined by the WHO, which includes dyspnea, hypoxia, more than 50% lung involvement on imaging, respiratory failure, shock, or ICU admission for organ failure [27]. The overall risk of severe/critical COVID-19 illness was higher in the hyperglycemia group than in the euglycemia group (OR = 2.08, 95% CI, 1.45–2.99, *I^2^* = 77.9%, *P*<0.001) (Figure 3). Although Begg’s test (*P*=0.133) did not reach statistical significance, Egger’s test (*P*=0.005) and dissymmetric distribution funnel plots (Supplementary Figure S2) indicated that publication bias may exist. Therefore, trim-and-fill test and sensitivity analysis were further conducted and our results were determined to be stable (Supplementary Figure S3). The results of the sensitivity analysis were not changed by excluding one included study at each time point (Supplementary Figure S5).

## Discussion

The COVID-19 pandemic has undoubtedly become a worldwide catastrophe in 2020 [28], and COVID-19 greatly threatens the health of people worldwide, consumes great amounts of medical resources, and stagnate the economy. Concerns about the clinical characteristics of COVID-19 patients with poor outcomes have been raised, and among them, hyperglycemia has been suggested to be related to the poor prognosis of COVID-19 patients in a meta-analysis [12]. However, considering that the previous meta-analysis included only eight studies and failed to demonstrate the causality between hyperglycemia and COVID-19, we further conducted this meta-analysis which focused only on hyperglycemia at admission and proved the predictive role of hyperglycemia at admission in COVID-19.

### Association between hyperglycemia at admission and COVID-19

Our meta-analysis included 16 high-quality studies and encompassed more than 6386 patients with COVID-19. Our findings demonstrably revealed that compared with COVID-19 patients in the control group, those in the hyperglycemia at admission group had an increased risk of mortality and severe/critical illness, implicating that hyperglycemia at admission may be an important predictive indicator of COVID-19 outcomes.

### Underlying mechanisms of the effects of hyperglycemia at admission on COVID-19

With regard to the underlying mechanisms of the impact of hyperglycemia at admission on COVID-19 outcomes that remain to be fully researched, there are several possible explanations that may account for the poor prognostic effect of hyperglycemia on COVID-19. First, the dysfunction of inflammatory cytokines and the immune system induced by hyperglycemia might play a role in the propensity for poor outcomes and mortality in COVID-19 patients. Patients suffering from severe COVID-19 have been observed to show higher levels of serum proinflammatory cytokines such as interleukin (IL)-6 (IL-6), IL-1 and tumor necrosis factor (TNF)-α, also known as ‘cytokine storm’ or ‘inflammatory storm’ [1,29–31]. Of note, IL-6, a major pro-inflammatory cytokine produced mainly by macrophage and T-helper 2 (Th2) cells, has been demonstrated to be significantly associated with the development or severity of COVID-19 in many studies [32–34]. It is known that IL-6 also plays a bad role in body health because it can damage DNA, lipids and DNA by aggravating oxidative stress [35]. Moreover, IL-6 could inhibit T-cell proliferation and B-cell differentiation to induce immunity dysfunction [29]. In particular, compared with IL-6 levels in COVID-19 patients with normoglycemia, higher levels of IL-6 in patients with hyperglycemia or elevated blood sugar were observed on admission [36]. Therefore, hyperglycemia may induce a burst of IL-6 to participate in the progress of cytokine storm to aggravate COVID-19 symptoms.

Second, hyperglycemia in severe COVID-19 patients has been demonstrated to down-7regulate the proportion of immune cells, including CD4^+^, CD8^+^ T cells and macrophages [37]. Consistent with this, lymphocytopenia is common in MERS patients with serious illness [38]. Similarly, up to 80% of severe COVID-19 patients have lymphocytopenia [39]. Moreover, low lymphocyte number (less than 0.6 × 10^9^/l) at admission has been demonstrated to be a risk factor for COVID-19 mortality [24]. COVID-19 patients with hyperglycemia tend to have lower lymphocyte counts than COVID-19 patients with normoglycemia. Therefore, we speculate that low innate immune defense resulted from decreased innate immune cells proportion may also account for the hyperglycemia induced mortality and severity of COVID-19.

In summary, high pro-inflammatory cytokines combined with low innate immune defense may contribute to the hyperinflammatory state in COVID-19 patients with hyperglycemia.

Additionally, angiotensin-converting enzyme 2 (ACE2) was confirmed to be the cellular receptor for COVID-19 infection and entry [40] and was reduced in COVID-19 patients along with concomitant cellular damage, respiratory failure and hyperinflammation [40]. Given that the renin–angiotensin system and ACE2 expression can be overactivated by hyperglycemia, poor glucose control in COVID-19 patients is likely to facilitate virus entry and infection [41]. Thus, the overexpression of ACE2 caused by hyperglycemia may be another reason for the poor prognosis of COVID-19 patients with uncontrolled blood glucose.

Last, abnormal blood coagulation function represented by d-dimer levels has been shown to worsen the disease condition with an increased death rate [42], and anticoagulation therapy has been utilized to improve the control of coagulation function in COVID-19 patients [43]. An observational study demonstrated that, compared with patients with well-controlled glucose levels, COVID-19 patients with poorly controlled glucose levels have increased levels of d-dimer [15]. Thus, we hypothesized that hyperglycemia may cause abnormal d-dimer levels and coagulation disorders, which in turn lead to the poor prognosis and high mortality of COVID-19 patients.

### Implications for practice and research

Considering that there is still no clear concept or conclusive evidence of the importance of blood glucose control in the prevention and treatment of COVID-19, our results definitely highlight the harmfulness of hyperglycemia at admission, which can be used as a clinical reference and has potentially important implications in clinical practice. On the research side, the underlying mechanisms of the effects of hyperglycemia on COVID-19 may be one of the important directions of future research in this field to improve COVID-19 outcomes.

Several limitations of our meta-analysis should be noted. First, publication bias was shown in the association between hyperglycemia at admission and complications of COVID-19. Second, another limitation was the unsolved heterogeneity in Figure 3 among the included studies. However, all the results of the included studies demonstrated that hyperglycemia at admission was positively associated with the severity of COVID-19, which indicates that our results should still be credible, although heterogeneity exists. Therefore, high-quality prospective studies are required to fill the evidence gap for the adverse prognosis of poor glucose control in COVID-19 progression.

### Conclusion

In summary, our research demonstrated that hyperglycemia may contribute greatly to the increased exposure of COVID-19 adverse outcomes and mortality. Strict glucose control is necessary during the treatment or prevention of COVID-19. Therefore, we recommend that glucose level screening is a medical necessity during the COVID-19 pandemic to improve the condition and prognosis of patients with COVID-19.

## Supplementary Material

Supplementary Figures S1-S5

## Data Availability

All data generated or analyzed during the present study are included in this published article.

## Abbreviations

ACE2: angiotensin-converting enzyme 2
CI: confidence interval
COVID-19: coronavirus disease 2019
FDA: the US food and drug administration
IL: interleukin
LOS: length of stay
MERS: middle east respiratory syndrome
NOS: Newcastle–Ottawa Scale
OR: odds ratio
SARS-CoV-2: severe acute respiratory syndrome coronavirus 2
